# Laparocopic ventral hernia repair with primary transparietal closure of the hernial defect

**DOI:** 10.1186/1471-2482-12-S1-S33

**Published:** 2012-11-15

**Authors:** Roberto Rea, Paolo Falco, Domenico Izzo, Maddalena Leongito, Bruno Amato

**Affiliations:** 1 Department of General Surgery, Clinica Mediterranea, Via Orazio, 2, 80122 Napoli, Italy; 2 Department of General Surgery, University “Federico II” of Naples, Via Pansini, 5 80131 Naples, Italy; 3 Department of General Geriatric, Oncologic Surgery and Advanced Technologies, University “Federico II” of Naples, Via Pansini, 5 80131 Naples, Italy

## Abstract

**Background:**

The treatment of ventral hernias is still a subject of debate. The affixing of a prosthesis and the subsequent introduction of laparoscopic treatment have reduced complications and recurrences. The high incidence of seromas and high costs remain open problems.

**Methods:**

At our Department between January 2008 and December 2011, 87 patients (43 over 65 years), out of a total of 132, with defects of wall whose major axis was less than 10 cm, or minor and multiple defects (Swiss-cheese defect) on an axis not exceeding 12 cm underwent laparoscopic ventral hernia repair (LVHR) with primary and transparietal closure of the hernial defect. Through small incisions in the skin we proceeded to close the parietal defect with sutures tied outside. Then the mesh was fixed as usual with double row of stitches and an overlap of 3-5cm.

**Results:**

In all patients, 43 of them elderly, surgery was successfully conducted. The juxtaposition of the edges of the hernial defect has not been time consuming and has not developed new complications. The postoperative course was uneventful, with discharge on the third day, except in 5 patients. Were observed only small gaps and not the formation of large seromas. There were no infections wall. We do not have relapses, but some small and asymptomatic solutions continuously up to 2 cm at the sonographic study. In elderly patients the absence of dead space and the feeling of greater stability of the wall, early mobilization and pain control have facilitated the post-operative course.

**Conclusions:**

The positioning of sutures transcutaneous is simple and effective, the reduced incidence of seromas and the greater stability of the wall suggest to adopt this procedure fully.

The possibility to close the margins of the defect may allow to change the size and setting of the mesh, since the absence of dead space allows to download physiologically tensions of the wall.

## Background

Ventral hernias, whether naturally occurring or the result of previous surgery, comprise one of the most common problems confronting general surgeons, with overall incidence between 2 and 13% [1-4]. The introduction of the anterior positioning of prosthesis has allowed to greatly reduce the recurrence. Laparoscopic ventral hernia repair (LVHR) was described on firsts by Leblanc in 1993 for all types of hernia [1-5]. This surgical technique has improved over the last decade and has proven to be an effective treatment option. With fewer wound complications, faster functional recovery and improved cosmesis has become a solution of choice in the treatment of incisional hernia [6,7]. However there are still some unresolved issues: a certain number of relapses, problems of fixation of the mesh, the choice of the mesh, and the incidence of seromas. Primary closure of the hernial defect is desirable, although technically complex, as shown by previous experience [6,8]. We report our experience (2008-2011) in the treatment of incisional hernias by laparoscopy in elderly patients with primary closure of the hernial defect with transparietal access.

## Methods

A total of 132 patients underwent LVHR at our department between January 2008 and December 2011, 63 aged over 65 years.

We chose as criteria for inclusion defects of wall whose major axis was less than 10 cm, or minor and multiple defects (Swiss-cheese defect) on an axis not exceeding 12 cm. 87 patients were included in the study (43 over 65), with an average age of 62 years (24-80) and an ASA between 1 and 3.

The patient is positioned supine with the arms alongside the body on an operating table tilting on all levels, to better expose the surgical field. The laparoscopic column is placed to the right of the patient for median defects, on the side of the defect if the same is lateral, with surgeons on the opposite side and trocar to draw a wide semicircle.

The initial peritoneal access is by a Verres needle puncture, 2 cm below the left costal margin in the midclavicular line (Palmer's point) [9,10]. Generally, three / four trocars and a 30° optic were used. Careful, often complex, adhesiolysis represents the first time. Then through incisions of 2 mm, made along the major axis of the hernial defect and on cutaneous projection of the same, spaced apart by about 2 cm, we proceed to the progressive approach of the edges of the hernia by placing sutures to X full thickness with a slow absorbable suture, resulting internally sutures spaced approximately 8 mm. A suture grasper closure device (Mediflex surgical products - Suture closure grasper device) allows a robust parietal crossing and easy maneuverability intracavitary.

Accurate defect measurement can be difficult. A sterile flexible ruler can be sized to fit through a trocar, and direct measurements can be taken internally. Lastly the mesh (Gore Dual Mesh ®) was rolled and inserted through the 10/12 mm port. The mesh was relaxed and hanging on the wall to the 4 cardinal points through the entire thickness and size suitable to ensure an over lap of 3-5 cm compared to the original defect. All precautions were taken to avoid contamination of the mesh with skin pathogens.

The mesh was fixed with a double row of absorbable straps (Ethicon Cincinnati USA. Securestrap TM). Drains are not used. The patients received intravenous ketorolac and tramadol for postoperative 24h to alleviate discomfort and pain, and if necessary Paracetamol 1000 mg 8-hourly. The antithrombotic protection was obtained with UFH 4000 IU per day.

A compressive dressing is maintained until the seventh post-operative day, to facilitate the consolidation of the plastic of abdominal wall, and an elastic band used for another 30 days, in patients with defects exceeding the 3cm. A VAS scale (1-10) was used to monitor the pain for 15 days. Mobilization occurred in the first day. The oral feeding was resumed on the first day only for the liquid and the second for solids. The discharge occurred on the third day, in the absence of hyperthermia with at least emission of flatus.

The patients were first followed up on the 7th post-operative day for dressing and stitches removal. They were subsequently followed up on 3 months post operatively, and yearly thereafter. During follow-up visits, a clinical examination and U.S. examination were performed to exclude recurrence of hernia or seromas.

## Results

Eighty-seven (43 over 65) patients were subjected to ventral hernia repair by laparoscopic approach with the primary closure of the hernial defect with external access. Run the operating was regular, sometimes complex for adhesiolysis, the mesh placement easier for the size of mesh not extreme (8-84 minutes, and 12-21 minutes respectively). The primary closure of the hernial defect has resulted in an additional expenditure of time (2-16 minutes). In the first 48 hours post-operative pain was mild (VAS 1-3) in 37 patients, moderate (4-6) in 48 patients and severe (7-10) in 2 patients (Table 1), however well controlled by analgesic therapy. The mobilization and channeling of recovery occurred in the first 24-48 hours while respecting the habitus, age, and the psychology of the patient, as well as the size of the hernial defect.

All patients had a regular course and discharged on the third day p.o., except 5 patients discharged on the fifth day due to fever (1), pain (3), slow bowel movements (1).

The parietal wound was regular in all cases without evidence of seromas, only modest absorption wall in 8 patients, regressed in a fortnight. Of these, 6 had major defects (> 6 cm) and 2 multiple defects. Over the next 15 days, the pain has always been mild (<3). In most cases, the pain was reported deep without affecting mobilization and ambulation. At ultrasound guidance in 15 days, 10 patients had moderate fluid collections (0.3 x 1.5 cm to 0.7 x4cm), mild illness. Only 1 patient confirmed the picture to 30 days. And thereafter were negative except for slight and episodic tenderness. To control to one year we observed ultrasonographically in 2 patients with defects of more than 6 cm, and 1 patient with multiple defects, the presence of small fascial defects of 2 cm asymptomatic and without commitment hernial.

## Discussion

Ventral hernias are defects of the anterior abdominal wall. They can be classified into congenital (epigastric, umbilical and Spigelian) and acquired (incisional) [1,11]. Incisional hernia is a frequent complication of laparotomies with overall incidence between 2 and 13% [1-4]. The development of a incisional hernia is usually due to a failure to heal or a late diastasis of the fascial planes. In the case of infection of the surgical wound, the incidence of incisional hernia increases up to 30% [12]. A just cause must be considered a disease and multiple multiorgan since, in relation to the seat and to the size of the defect, can interfere with the dynamic respiratory, vascular or other viscera [13].

A significant reduction in the number of relapses was obtained with the introduction of mesh that reinforced the fragile scar tissue. LVHR (Leblanc 1993) has gained tremendous popularity over the last decade for fewer wound complications, faster functional recovery and improved cosmesis, and can be considered to be the procedure of choice in the treatment of ventral hernias. This procedure applies the principles derived from the Rives-Stoppa repair [1,5,14,15] with modifications in the technique for mesh placement. The prosthetic mesh is used to reinforce the fascial defect and the anterior abdominal wall intraperitoneally, thereby minimizing the complications associated with extensive dissection of the retro-rectus extraperitoneal space [1,16-18]. However, we agree with the statement that the ideal treatment should aim to restore the integrity of the abdominal wall and redistribute the intra-abdominal pressures and tensions, especially in hernia of medium and large size (13). And if this is not possible in large and cronic parietal defects, in most cases must be a precise objective. So, the laparoscopic lysis of adhesions with the reduction of the hernial content without preparation of the wall layers, the prosthetic reinforcement, accompanied by the combination of direct hernia margins appear benchmarks for optimal surgical management and subsequent functional recovery. In our current study, we modified the standard LVHR whether to evaluate the incidence of recurrence, of seroma and postoperative pain could be minimized further.

Previous research [1,8] has proposed the use of a straight needle or half circle with difficult maneuvers laparoscopic, knotting complicated, uncertain and incomplete approach the edges of the parietal defect. The same reduction of the room peritoneal which favored the approach margins and hindering the passage of the needles and maneuvering of knotting. The placement of sutures transparietal is certainly less risky, more wall thickness determined and knotting, outdoor, easier and above all stronger. In contrast to other devices on the market, the caliber of this needle is sufficient to withstand the pressures of inclination which are often necessary and the exposure of the arms facilitates the grip and release of the suture without any further solicitations. The realization of points to X at an average distance of 8 - 10 mm enables to obtain a solid combination of the margins of the defect. The knotting is easier for the possibility of reducing the pneumoperitoneum during these maneuvers with reduced risk of injury of the underlying viscera and for the progressive approach that reduce the tensions of the centrifugal wall. This reinforcement allows the mesh to better integrate itself into the wall, in the absence of tension, without entrust to the double row of staples the full responsibility for the establishment.

The post-operative pain showed a slight increase in the first 48 hours (Table 2) compared to previous observations of our cases that had not been achieved with the median approach, however well-controlled by analgesic therapy. This could be motivated by the fact that the reduction of the abdominal tensions obtained in laparoscopy, due to the absence of juxtaposition of the muscle and the fascial, is partially revived in our experience, even if to a lesser extent compared to the preparation layers by front access.

The choice of an overlap of 3-5cm is generally shared, so in this first phase we have preferred to keep the size of the mesh established before the closure of the defect. In the face of demonstrated effectiveness of primary closure of the defect is conceivable containment of mesh size with overlap calculated from the midline. This would allow an introduction and positioning easier, a considerable saving on the cost of the mesh and the safeguards to his fixation.

Restore muscle continuity is certainly preferable to the remaining dead space, except as necessary to excessive tension that could result in excessive pain and reduced respiratory capacity. It can be hypothesized that, by closure of the defect, abdominal wall integrity is restored, leading to equalized pressure and tension across the abdominal wall and intra-abdominally placed mesh. Then it would seem that there was no significant correlation between transfascial sutures and postoperative pain. The suture of the defect reduces the "dead space" preventing the formation of seromas [19,20] .In our experience, the risk of seroma is strongly contained by the lack of room remaining. Were observed only small residual pockets and asymptomatic at the ultrasound control, probably due to the persistence of the peritoneum in the sac. The literature reports 14.5% of formation of seromas in the absence of closure of the defect [19]. Le Blanc [21] highlights the problems of prosthesis fixation with external or internal sutures. This enables our proposition of partially overcome the problems of fixation of the mesh, relegating the function of the prosthesis and its stabilization at only structural reinforcement, as well as already ascertained in other districts.

The stabilization of the defect appears to be effective even in our initial cases, further reducing the recurrence rate. Small, asymptomatic, sagging highlighted with postoperative ultrasound not have affected the stability of the mesh. Further developments may be represented by the possibility to fix with the only fibrin glue or other adhesive systems the mesh, which is immediately solicited without primary closure appear less efficient. [22-24].

## Conclusions

The primary closure of the hernial defect allows better to reinforce the wall, to reduce the dead space and the possibility of formation of seromas.

Its realization transparietal appears semple and safe, inexpensive of time.

The positioning of the mesh is easier and safer and reduced wall stress.

Possible evolutions are represented by the possibility of reducing the titanium o aborbable tacks and also the size of the mesh, with significant cost savings.

## List of abbreviations used

LVHR: Laparoscopic Ventral Hernia Repair

## Competing interests

The authors declare that they have no competing interests.

## Authors' contributions

RR: conception and design, interpetration of data, given final approval of the version to be published; PF: acquisition of data, drafting the manuscript, given final approval of the version to be published; DI: acquisition of data, drafting the manuscript, given final approval of the version to be published; ML: acquisition of data, drafting the manuscript, given final approval of the version to be published; BA: conception and design, critical revision, given final approval of the version to be published.
